# Evolutionary Origins of Genomic Repertoires in Bacteria

**DOI:** 10.1371/journal.pbio.0030130

**Published:** 2005-04-05

**Authors:** Emmanuelle Lerat, Vincent Daubin, Howard Ochman, Nancy A Moran

**Affiliations:** **1**Department of Ecology and Evolutionary Biology, University of ArizonaTucson, ArizonaUnited States of America; **2**Department of Biochemistry and Molecular Biophysics, University of ArizonaTucson, ArizonaUnited States of America; University of TexasUnited States of America

## Abstract

Explaining the diversity of gene repertoires has been a major problem in modern evolutionary biology. In eukaryotes, this diversity is believed to result mainly from gene duplication and loss, but in prokaryotes, lateral gene transfer (LGT) can also contribute substantially to genome contents. To determine the histories of gene inventories, we conducted an exhaustive analysis of gene phylogenies for all gene families in a widely sampled group, the γ-Proteobacteria. We show that, although these bacterial genomes display striking differences in gene repertoires, most gene families having representatives in several species have congruent histories. Other than the few vast multigene families, gene duplication has contributed relatively little to the contents of these genomes; instead, LGT, over time, provides most of the diversity in genomic repertoires. Most such acquired genes are lost, but the majority of those that persist in genomes are transmitted strictly vertically. Although our analyses are limited to the γ-Proteobacteria, these results resolve a long-standing paradox—i.e., the ability to make robust phylogenetic inferences in light of substantial LGT.

## Abstract 2

Lateral gene transfer, rather than duplication, is responsible for most gene diversity present in gamma-Protobacteria; however, these genes are then vertically transmitted and have little impact on gene phylogenies

## Introduction

The complexity and coordination of cellular functions are remarkable in view of the disparate histories of the genes that make up contemporary genomes. In eukaryotes, new genes arise primarily through the duplication of existing genes [1,2,3,4], while some ancestral genes are inactivated or eliminated over time. In contrast, prokaryotic genomes undergo substantial rates of gene acquisition from foreign sources [5], as well as duplication and loss of existing genes. Thus, if we consider the gene repertoire of a particular bacterial cell, some genes have been transmitted vertically for very long periods of time, perhaps from the time of the common ancestor of all cellular life-forms, whereas other genes were acquired or generated at various points in the history of the lineage, including some very recently.

Although the role of vertical transmission and horizontal transfer are both well documented, as yet, we have no comprehensive, quantitative picture of the genome-wide history of gene gain and loss over time for any particular prokaryotic group. The availability of many complete genome sequences of bacteria presents the possibility of tracing the history of individual genes within evolving lineages by identifying the points at which genes originate through acquisition or duplication, and the points at which genes are lost. The resulting picture would address several outstanding questions and paradoxes concerning bacterial genomes. For example, if there is a robust estimate of the cell (or organismal) phylogeny for a set of lineages, can we identify the events of gene acquisition, duplication, and loss that lead to the current gene repertoires of individual cells? Is the incidence of gene acquisition ongoing or episodic, and do acquired genes come from very close relatives or from distant sources? Of acquired genes, what types and what proportion become permanently installed within descendant genomes, and which are lost?

It is clear that gene duplication, gene loss, and gene transfer all impact bacterial genomes; but the relative contributions of each remain controversial [6,7,8,9,10,11,12,13]. The situation is confounded by the fact that, in bacteria, the presence of two or more homologous sequences within a single genome might reflect the acquisition of a gene copy from a foreign source rather than the duplication of a resident gene. In the absence of further analysis, such homologs cannot be confidently described as paralogs (or duplicates) [14] or as xenologs (acquired via horizontal transfer) [15], and we propose the term “synologs” as an agnostic name for homologs within a genome arising from either process. Distinguishing the origins of synologs within genomes allows both the accurate dissection of gene families and the full reconstruction of events responsible for the contents of cellular genomes.

Here we investigate the full protein-coding gene repertoires within the γ-Proteobacteria, a group chosen because the large number of fully sequenced genomes, combined with their well-supported phylogenetic relationships, allows us to trace the origins of new genes in organisms that differ widely in their gene inventories (ranging from 564 protein-coding genes in Buchnera aphidicola to 5,540 in Pseudomonas aeruginosa) [16]. This group is an ancient bacterial phylum, at least several hundreds of million years old, based on the sequence divergence within the group [17] and on its containing at least one ancient subclade (*Buchnera*) that has cospeciated with hosts for over 100 million years. Available genome sequences include species displaying diversified lifestyles and subject to varying degrees of gene acquisition [5,6,18,19,20].

By assessing the history of every gene family, we find that gene acquisition is a major factor contributing to genomic diversity of these bacteria, but that, paradoxically, they rarely exchange genes. In addition, duplication appears to have played a secondary role in the evolution of gene repertoires, as multigene families are scarce and a substantial fraction of the genetic redundancy observed in genomes is better explained by gene acquisition from a distant source. These results support the view that bacterial genomes evolve mainly by incorporation of completely novel genes rather than by intragenomic duplication or by replacement of resident genes with distant homologs.

## Results

Having defined all gene families of homologs present in 13 sequenced γ-proteobacterial genomes [16], we partitioned them according to their distribution among species and their incidence of synology. We examined the congruence of each of these families with the organismal phylogeny using maximum-likelihood (ML) tests, and we provide two estimates (one stringent and one permissive) of the number of lateral gene transfers (LGTs) found in these families (Figure 1).

**Figure 1:**
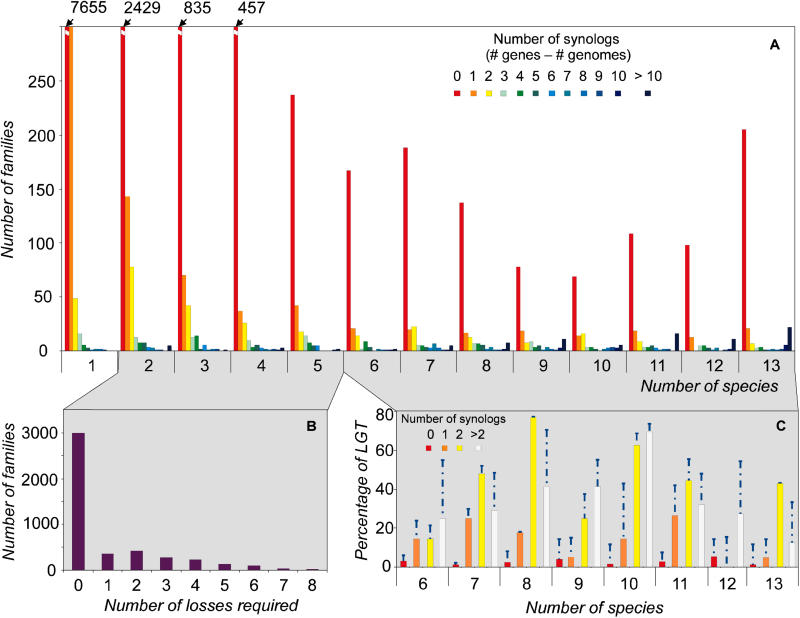
Distribution of Gene Families and Occurrence of LGT in γ-Proteobacteria (A) Numbers of species and numbers of synologs corresponding to the 14,158 gene families. Single-copy gene families (red bars) comprise the large majority of the families. The numbers of families in categories exceeding 300 members are displayed on top. (B) Losses required to reconcile gene distribution with organismal phylogeny [16] for gene families represented in fewer than six species. For each family, we inferred an initial acquisition event in the most recent ancestor of the species containing a gene from the family and tallied the minimum number of independent events of loss required to explain the phylogenetic distribution. Most distributions can be explained without invoking multiple gene losses, supporting the hypothesis of a single acquisition. (C) Percentage of families containing fewer than three synologs (# gene copies − # genomes = 0, 1, or 2) showing evidence of LGT by the method described in Lerat et al. [16] and Figure 2. Boxes represent the conservative estimate of LGT and dashed bars represent the corresponding permissive estimate (see text). For families containing additional synologs (white bars), it was not practical to apply the same method; instead, we built neighbor-joining trees (see Materials and Methods).

### Single-Copy Genes

Among single-copy genes present in six to 12 genomes (Figure 1A: red bars in categories 6–12), 1%–5% display statistically supported incongruence with the organismal phylogeny (red bars, Figure 1C), a low incidence in view of the high frequency of acquired genes in some of these genomes. The more permissive estimates (dashed lines in Figure 1C) imply that up to 15% of these families with no synologs may have experienced LGT. This low rate of LGT in gene families not universally distributed was statistically indistinguishable from that of genes present in all 13 genomes (χ^2^ test, *p* > 0.1). The absence of these gene families from one or more genomes could result either from presence in the ancestor followed by loss in some lineages, or from absence in the ancestor followed by acquisition from a distant source in a descendant lineage. Although this implies ongoing loss and acquisition of genes, our results indicate that even genes initially acquired from distant sources are rarely transferred subsequently among lineages of γ-Proteobacteria.

### Occurrence and Source of Synology in Bacterial Genomes

The sizes of gene and protein families in bacterial genomes have previously been shown to follow a power law distribution [21,22,23,24]. Within the γ-Proteobacteria, we find that, overall, very few gene families contain synologs, as evident in the low frequencies of families in which members outnumber genomes (Figure 1A). When present, synology might be expected to be associated with LGT, given that gene acquisition is itself a possible source of synologs within a genome. Applying tests of phylogenetic congruence to distinguish between intragenomic duplication and LGT (Figure 2), our stringent estimate is that LGT can be implicated as the cause of synology in 22% (51) of the 231 families in which gene copies outnumber genomes by one or two. In families with one or two synologs, LGT occurs at significantly higher frequencies than in families of similar species distribution but lacking synologs in both the stringent and permissive tests (*p* < 0.0001; see Figure 1C). This difference between the amount of LGT in families with and without synologs was also found to be significant using the more permissive estimates of LGT. In only 3% of the 231 families was the conflict with the organismal phylogeny not confined to synologs (see last column in Figure 2) and more readily explained as due to a gene transfer elsewhere in the tree. Hence, most of the incongruence observed in families containing synologs can be explained by a single transfer event of a gene for which a homolog was already present in the recipient genome. In Table 1, the proportion of gene families showing evidence for LGT is classified according to their functional categories. Most families are of unknown function (and are listed as “hypothetical proteins”); however, several have been assigned to broad functional categories, such as “energy metabolism,” “cellular processes,” “transport and binding proteins,” and “amino acid biosynthesis.”

**Figure 2:**
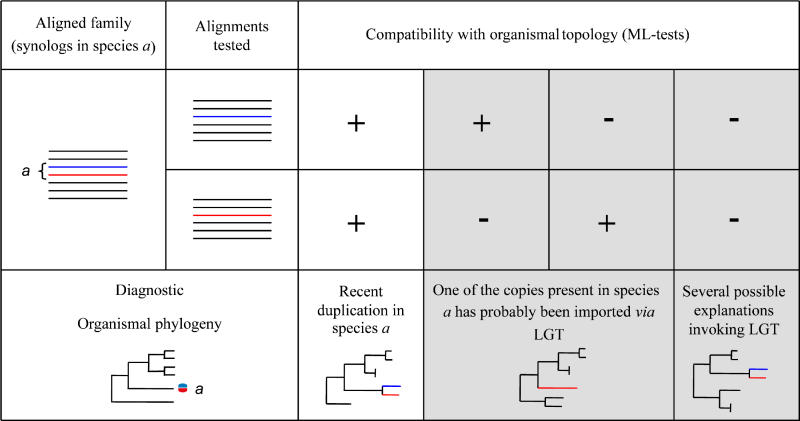
Testing for LGT and Duplication as Sources of Synology The illustrated case is for families with a single synolog, that is, in which one genome contains two gene copies. We tested two alignments, each retaining one of the two copies (red or blue), against the reference organismal phylogeny [16]. When both alignments agreed with the reference tree (+/+), the synology could be attributed to recent intragenomic duplication, whereas in cases of phylogenetic incongruence of one of the alignments (+/− or −/+), LGT of one synolog was invoked. If both alignments rejected the reference tree (−/−), the family was considered as containing one or several LGT events. Tests of LGT were conducted similarly for families with an additional synolog (one genome with three synologs or two genomes each with two synologs). In such cases, each possible alignment containing a single copy per genome was tested. In addition to these tests, all family trees were inspected to confirm diagnoses of LGT.

**Table 1:**
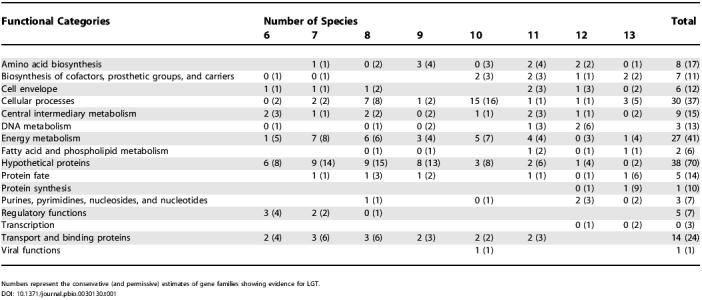
Numbers of Gene Families Showing Evidence for LGT

Numbers represent the conservative (and permissive) estimates of gene families showing evidence for LGT

For families with larger numbers of synologs, the direct comparison of gene trees with the reference topology also indicates high levels of LGT (up to 60%), although its inference is less definite due to uncertainties in reconstructing complex histories of multiple gene gains and losses. Families with three or more synologs are few in number (<2% of the 14,158 families) but include some instances of ancient gene duplication preceding the diversification of lineages.

### Phylogenetic Signal and the Evidence for Vertical Inheritance

In tests for phylogenetic congruence, alignments that do not reject the reference organismal phylogeny are usually interpreted as reflecting vertical inheritance. However, such results, that is, the absence of a significant difference from the reference topology, can also be caused by phylogenetically uninformative alignments. Such problems are most likely for extremely divergent sequences, for which alignment and phylogenetic inference procedures are prone to failure, or for very short sequences, which may lack sufficient numbers of informative sites. Our gene families were constructed so as to exclude extremely divergent sequences, leaving the possibility that short genes are the most problematic ones. But, in our tests, there was no significant difference (*p* > 0.2) in the incidence of LGT among genes of different size categories, implying that lack of sufficient information was not a primary reason for failing to reject the reference topology. Furthermore, to explain the result whereby families with synologs display more LGT than those without, one would need to hypothesize that the lack of phylogenetic signal is restricted to families without synologs. However, the difference in the frequency of LGT between the families with and without synologs remains evident in each of the size categories (see Figure 1C). Cumulatively, these analyses indicate that our tests of phylogenetic incongruence have sufficient signal to infer vertical inheritance and are not affected by gene size.

### Genes with Very Limited Phylogenetic Distributions

About half of the gene families (7,655 of 14,158; Figure 1A) contain a single member confined to a single genome. The fraction of these genes in a genome varies as a function of the local phylogenetic sampling (from <5% in Yersinia pestis CO92 to 40% in *Pseudomonas*) and of the evolutionary constraints on a genome (with few such genes in the highly reduced genomes of the endosymbionts *Buchnera* and *Wigglesworthia*). Two reasons may account for the exclusion of these genes from other families: first is the possibility that the threshold for delineating families was too restrictive and did not allow inclusion of distant homologs. In this case, there is a chance that a very quickly evolving gene might be assigned to its own, single-member family. Alternatively, genes that are unique to genomes may represent recent acquisitions from distant sources outside of the γ-Proteobacteria. To discriminate between these situations, we conducted a blastp search on each of the unassigned proteins that were confined to one γ-proteobacterial genome on the database containing all proteins present in sequenced bacterial genomes (EMGLib release 5 [25]).

This analysis, in which the cutoff for protein matches is based on e-values rather than on an empirically determined percentage of the maximal bit score, provided evidence that the majority of the single-member gene families within γ-proteobacterial genomes could be attributed to LGT. Only 17.5% of the proteins unique to a single genome had matches in other γ-proteobacterial genomes. These potentially represent quickly evolving genes that were originally excluded from protein families because of insufficient similarity. In contrast, 40% of the unique proteins gave hits in organisms outside of the γ-Proteobacteria, a distribution that will most likely arise by LGT between distantly related lineages. The remaining 42.5% of the single-member gene families correspond to orphan open reading frames (ORFans), that is, genes that have no homologs in the current databases.

Alternatively, this last category could result from the misannotation of genome sequences. However, a recent study of ORFans in Escherichia coli demonstrated that most encode functional proteins [26]. ORFan genes tend to be short and enriched in A/T nucleotides when compared to the rest of the genome, features that suggest that they originated in parasitic elements, such as bacteriophages [26]. An analysis of the base composition of the sets of unique genes in the γ-Proteobacteria demonstrate that in all genomes (with the exceptions of *Buchnera, Wigglesworthia,* and *Haemophilus,* each possessing few, if any, unique genes), ORFans are significantly biased toward A+T at the third codon positions when compared with other genes in the genome (averaging a 5% difference in A+T contents; *p* < 0.05). This result is consistent with the hypothesis that these genes, which have no matches in current databases, have been recently acquired from bacteriophages, whose diversity is largely unsampled and unknown [27]. Therefore, the prevalence of gene families restricted to one or a few genomes (Figure 1A) supports gene acquisition as a principal source of new genes in this group of bacteria.

For families containing single members in four or five genomes, ML tests supported phylogenetic congruence for nearly 100% of cases (results not shown). However, this high degree of congruence could reflect, in part, the large number of gene families shared by closely related genomes (e.g., the two *Yersinia* or the two xanthomonads). To further evaluate those families (with and without synologs) present in two to five genomes, we enumerated the gene losses required to explain the phylogenetic distribution of the family under the assumption of no LGT following a single initial appearance in a lineage (Figure 1B). For 74% of these families, the occurrence of homologs among the taxa can be explained as a single acquisition by their common ancestor, followed by vertical inheritance with inference of, at most, a single subsequent loss.

Cumulatively, the phylogenetic evidence (for gene families present in six or more genomes) and the distributional evidence (for gene families present in fewer than six genomes) indicate that high levels of foreign gene acquisition have introduced the majority of genes of γ-proteobacterial genomes, but that this gene acquisition has little impact on gene phylogenies within this group. Massive gene uptake does not cause phylogenetic inconsistencies because (i) acquired genes come from sources outside of this group, (ii) they rarely have homologs within the recipient genome, and (iii) subsequent to their initial acquisition, genes tend to be vertically transmitted.

### Extent of Gene Origination and Acquisition among Taxa

The incidence of LGT varies enormously among the lineages included in our tree. For instance, in addition to possessing a very large number of unique genes, the genome of P. aeruginosa contains numerous genes from families whose phylogenetic distribution can only be explained by a very large number of gene losses in other lineages or by LGT (Figure 1B). This species shares numerous genes with one other distantly related species (e.g., 28 with E. coli and 28 with *Salmonella enterica,* each of which would require the inference of five independent gene losses under the assumption of a single initial acquisition) or with a distant sister pair (43 with *Escherichia* + *Salmonella,* and 50 with the two *Yersinia,* corresponding each to a scenario invoking four gene losses). Additionally, P. aeruginosa is the only species in this group for which instances of LGT for single-copy, broadly distributed genes have been detected [16]. At the other extreme, the endosymbiotic species (*Buchnera* and *Wigglesworthia*) show virtually no evidence of gene acquisition.

## Discussion

Previous attempts to reconstruct the history of gene repertoires in bacteria have examined gene distributions on a species phylogeny [9,13,28]. But ignoring the relationships among homologs will lead to incorrect assessments of the relative contributions of gene gain, loss, and duplication to genome inventories. For example, if a gene has spread widely through LGT, an analysis based on gene occurrence would conclude that this ubiquitous distribution resulted solely from vertical inheritance. Moreover, such methods cannot distinguish between LGT and duplication as the origin of synology and therefore provide a distorted view of the extent of duplication in bacterial genomes. Only by evaluating the evidence for concordance between the gene phylogenies and the organismal phylogeny is it possible to trace the history of gain, loss, and duplication affecting each gene family.

It was previously shown only about 200 single-copy genes are shared by these genomes [16] and that only 1% of these broadly distributed genes display statistically supported evidence of LGT. However, most of these genomes contain several thousand genes, indicating that the majority of genes in the genome were not present in the ancestor to all γ-Proteobacteria and that they originated either through LGT or by duplications as lineages diversified. By conducting an exhaustive phylogenetic analysis of all genes present in completely sequenced γ-proteobacterial genomes, we have evaluated the factors responsible for altering gene inventories and contributing to genomic innovation. It has long been recognized that duplication and LGT contribute to the genome composition of evolving bacterial lineages and, in particular, of lineages in the γ-Proteobacteria [5,29,30,31], and we provide a quantitative assessment of the roles of these processes on a genome-wide scale.

An enormous incidence of gene acquisition is suggested by the large number of genome- or clade-restricted gene families, but beyond their initial acquisitions, few gene histories conflict with the organismal tree. Our results show that most acquired genes lack homologs in the recipient genome and in other γ-Proteobacteria. Therefore, most of the genes present in contemporary genomes have arisen from distant sources. Although these genes may have been transmitted from unrelated cellular organisms, recent work revealing the previously overlooked diversity of bacteriophages [27,32] and their probable role in bacterial evolution [26,33] suggest that they have contributed significantly to the evolution of bacterial gene repertoires.

Traditionally, high levels of LGT have been considered to be incompatible with a tree-like representation of bacterial evolution. However, the diversity of gene families unique to single genomes indicates that the pool of available genes is very large, allowing the rate of gene acquisition to be both high for a genome and very low for a particular gene. Interestingly, there is no evidence that genes with narrower phylogenetic distributions were more likely to undergo LGT, suggesting that the essentiality of a gene, as denoted by its universal presence among species, is not a predictor of its propensity for LGT. Hence, once acquired, most genes appear to strictly follow the organismal phylogeny.

Whereas in eukaryotes, most multicopy genes arise from duplications, we find that LGT underlies a substantial proportion of the cases of synology in bacterial genomes. But, overall, synology is rare among gene families. Because duplicates are only rarely retained in bacterial genomes for long periods of time, hidden paralogy, that is, the differential loss of paralogs in independent lineages, is an unlikely explanation for phylogenetic incongruence. The overall paucity of families with synologs and their association with high rates of LGT indicate that duplications are not a major mechanism for diversifying functions in these bacteria. Although duplications play an important role in the short-term adaptation of bacteria [29,30], only a few duplicated genes are retained and subject to selection for diversifying functions. The fixation of duplicates requires the gradual evolution of sequence changes conferring differences in expression or function, whereas genes arriving through LGT are likely to be operationally distinct from those already present in a genome and, thus, immediately able to contribute unique functions and to be maintained in the genome by selection.

The large number of genes that are confined to a single genome indicates frequent gene acquisition in this group of bacteria. In contrast, substantially fewer genes are distributed in families present in more than one proteobacterial genome. Therefore, based on the distributions of gene families and on the abundance of genes confined to a single genome, recently acquired genes are lost most readily. This implies that genes are continuously integrated into the genomes but rarely persist long enough for hosts to diversify [31,34]. Although a few such genes could be present in multiple species, but quickly evolving and unrecognizable due to loss of sequence similarity, this situation cannot apply widely given the close relationships of some of the genomes [26]. Rather, most genes confined to a single genome reflect recent acquisition from a source outside of the sampled γ-Proteobacteria.

Cumulatively, the picture emerging from these studies is that bacterial lineages are constantly subjected to the input of new genes from a large available pool. Conversely, resident genes are continually lost. As a result, genomes contain sequences that have been resident in a particular lineage for very different durations (Figure 3). The extent of gain and loss can vary widely among lineages: among the γ-Proteobacteria, P. aeruginosa is at one extreme and contains a very heterogeneous assemblage of genes with distinct histories and varying widely in persistence, whereas *Buchnera* comprises genes with very long evolutionary histories within the cell lineage and essentially no recently acquired sequences. The coordination of complex networks of cellular functions is all the more remarkable given that the genes within a genome lack a cohesive history together.

**Figure 3:**
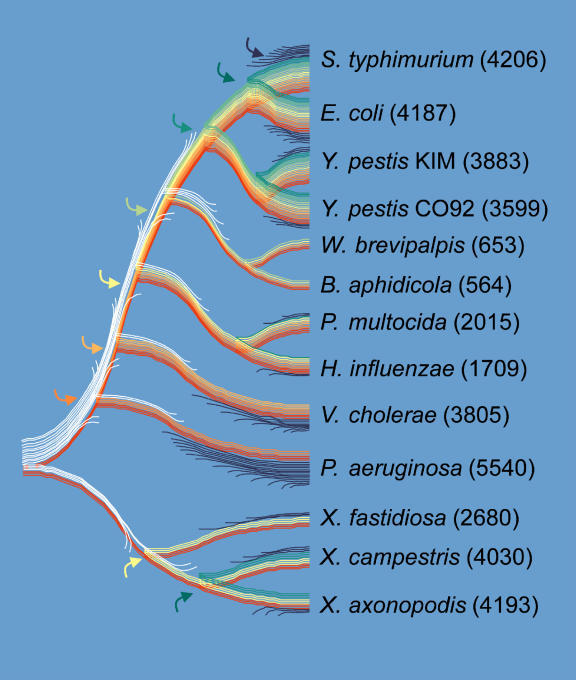
LGT and Genome Evolution in γ-Proteobacteria Only a small proportion of genes have been retained since the common ancestor of γ-proteobacteria (in red). Under the assumption that ancestral and contemporary genome sizes are similar, most of the genes present in this ancestral genome (in white) have been replaced by nonhomologous genes (yellow to green), usually via LGT from organisms outside of this clade. Once a new gene is acquired, its transmission follows vertical inheritance. The abundance of genes unique to a species (in blue) indicates that these bacteria (with the exception of the endosymbionts) constantly acquire new genes, most of which do not persist long-term within lineages. (Numbers of protein-coding genes, excluding those corresponding to known IS elements and phages, are in parentheses for each genome).

Our results, based on the distributions and phylogenies of all genes of a set of related genomes, provide a context for understanding several findings that previously seemed contradictory: extremely high levels of LGT [5], congruence among gene trees at various depths within bacteria [6,16,35,36], and general agreement of sequence-based gene trees with phylogenies based on genome contents [37,38]. We focused on the most intensively sequenced bacterial clade: as more genomic sequence data become available, similar approaches can be applied to determine if genome contents evolve in the same manner in other groups.

## Materials and Methods

### 

#### Defining gene families

To investigate the history of all protein-coding genes, we defined all gene families present in the following γ-Proteobacteria: E. coli K12 [39], B. aphidicola APS [40], H. influenzae Rd [41], Pasteurella multocida Pm70 [17], S. enterica serovar Typhimurium LT2 [42], Y. pestis CO-92 [19], Y. pestis KIM5 P12 [43], Vibrio cholerae (chromosomes I and II [44]), Xanthomonas axonopodis pv. *citri* 306 [45], X. campestris [45], Xylella fastidiosa 9a5c [46], P. aeruginosa PA01 [47], and *W. glossinidia brevipalpis* [48]. Protein sequences from complete genomes were retrieved from GenBank [49] and filtered to remove proteins annotated as insertion sequences or as bacteriophage sequences. Accession numbers for these genomes can be found in the Accession Numbers section of this paper.

Homologous genes (and resulting gene families) were defined using a cutoff for the degree of similarity among proteins reflected in the blastp bit scores [50]. The procedure for defining gene families was described in Lerat et al. [16] and is briefly summarized as follows: first, a bank containing all annotated protein sequences from all included species was queried with all the proteins in each of the genomes via blastp, such that all proteins were searched against both their resident genome proteins and those from the other species. To establish the threshold for grouping genes into a family, we examined the distribution of the ratio of the bit score to the maximal bit score (i.e., protein match against itself) based on that observed for the proteins of E. coli compared against proteins of the other genomes. In each case, there is a bimodal distribution, with a first peak of low similarity values, which is constant among comparisons and represents random matches, and a second peak of higher values, which varies from one comparison to another and therefore probably represents true homologs. The height of the second peak varies according to the number of gene family constituents and can range from one, for single member families, to hundreds. The two phases of the distribution are partitioned at approximately 30% of the maximal bit score, and thus proteins having bit score values ≥ 30% of the maximal bit score were considered homologous and members of the same gene family.

Genes were assigned to families by a simple link rule such that if gene A matches gene B, and gene B matches genes C, then all three are grouped into the same family. Comparisons among the families resolved after applying different thresholds (10%, 20%, 30%, or 40% of the maximal bit score) revealed that the 30% cutoff maximized the number of families containing genes from all 13 species, indicating that this criterion is optimal for the interspecific identification of homologous sequences. (Information about the distribution and constituents of gene families is available upon request from the authors.)

#### Gene origins and ancestries

Of the 14,158 gene families, 205 families are present as exactly one copy in each of the 13 genomes, and previous work has established that 99% (203) of these single-copy, widely distributed gene families are consistent with a single phylogeny, as expected if they share a history of vertical transmission through the replicating cell lineages [16]. This reference phylogeny provides a scaffold upon which the ancestry of every member of every gene family could be examined. To investigate how each gene originates within a genome and how gene families are generated, all protein-coding genes within each family were subjected to phylogenetic analysis.

Although strong evidence of LGT can be gained by a phylogenetic approach, several factors, including the sensitivity of the tests employed and the varied causes of phylogenetic incongruence (such as hidden paralogy or long branch attraction, besides LGT), can confound the interpretation of such analyses. Therefore, we estimated the frequency of LGT in gene families by both stringent and permissive approaches. The conservative estimates rely upon the analysis of four different ML tests of phylogenetic congruence and the visual inspection of the trees and alignments for each family. In this case, we require that at least three tests support phylogenetic incongruence and that this incongruence not be explicable parsimoniously by hidden paralogy or ambiguous alignments. In the permissive estimates, LGT is inferred when at least one out of the four tests supported phylogenetic incongruence and when the tree needed more than two independent gene losses to be explained by hidden paralogy.

Gene families differ in their distribution among the sampled genomes and in the numbers of members per genome, and we considered the following cases:

#### Families without synologs

We first focused on gene families that contained no synology (i.e., the number of genes equals the number of genomes in which family members are found) and whose members are present in at least six of the genomes considered. Sequences were aligned using ClustalW version 1.83 [51], and the best ML tree was inferred using proml from the PHYLIP package version 3.6 [52] with the JTT model of amino acid change [53] and a model of heterogeneity of evolutionary rates among sites (α parameter estimated from the dataset on the best tree, using Tree-Puzzle 5.1 [54]). The likelihood of this tree was then compared to the reference species phylogeny [16], using the different ML tests (Shimodaira-Hasegawa test [55], the one- and two-sided Kishino-Hasegawa tests [56,57], and the expected likelihood weights [58]) implemented in Tree-Puzzle 5.1 [54] with a confidence interval of 5%. LGT was inferred from the results of these different tests and by visual inspection of the tree and alignment for each family.

#### Families with synologs

In cases where a gene family contained one or two synologs (i.e., # species < # genes ≤ # species + 2), we addressed whether synology arose from LGT or from intragenomic duplication by analyzing all possible combinations of genes from an alignment but including only one gene per species via individual ML tests (see Figure 2 for an explanation of the case with one synolog). When the tree including a particular synolog was incongruent with the reference species tree, we considered that synolog as potentially arising from LGT. This diagnostic was subsequently confirmed by analyzing the tree based on all gene family members applying the procedures (described below) used for families containing more than two synologs. In such cases, LGT was inferred when the synology could otherwise be explained only by a scenario invoking at least three independent gene losses.

Because procedures that reconstruct all possible phylogenies using individual synologs are difficult to interpret when numerous synologs are present, the ML tests were not applied to families with more than two synologs. The number of such families was small, which enabled us to infer cases of LGT by inspection of tree topologies. For each family containing multiple synologs, a tree based on the whole family was built with “Neighbor” using a distance matrix obtained from protdist (JTT model of amino acid change [53]) from the PHYLIP package version 3.6 [52]. Distances were computed under the γ-based method for correcting the heterogeneity of rates among sites with the α parameter obtained from the dataset on the best tree, using Tree Puzzle 5.1 [54].

#### Families present in few species

For gene families present in fewer than six genomes, ML analyses either are not possible (when family members are present in fewer than four species) or might overestimate congruence (when pairs of very closely related genomes are included, such as the two *Yersinia* or the two xanthomonads). To further evaluate the incidence of LGT in gene families distributed in two to five genomes, we inferred an initial acquisition event in the most recent ancestor of the species containing a homolog and tallied the minimum number of independent events of loss required to explain the phylogenetic distribution. Families requiring the inference of zero, one, or two losses can most readily be interpreted as vertically transmitted following their origin in the shared ancestor. In contrast, families requiring inference of many losses would be most reasonably interpreted as having undergone multiple acquisition events from outside sources or transfer between lineages of γ-Proteobacteria.

## Supporting Information

### Accession Numbers

The GenBank (http://www.ncbi.nlm.nih.gov/Genbank/index.html) accession numbers for genomes discussed in this paper are Escherichia coli K12 (NC 000913), Buchnera aphidicola APS (NC 002528), Haemophilus influenzae Rd (NC 000907), Pasteurella multocida Pm70 (NC 002663), Salmonella enterica serovar Typhimurium LT2 (NC 003197), Yersinia pestis CO-92 (NC 003143), Yersinia pestis KIM5 P12 (NC 004088), Vibrio cholerae (NC 002505 [chromosome I] and NC 002506 [chromosome II]), Xanthomonas axonopodis pv. *citri* 306 (NC 003919), Xanthomonas campestris (NC 003902), Xylella fastidiosa 9a5c (NC 002488), Pseudomonas aeruginosa PA01 (NC 002516 [47]), and *Wigglesworthia glossinidia brevipalpis* (NC 004344).

## Abbreviations

LGT: lateral gene transfer
ML test: maximum-likelihood test
ORFans: orphan open reading frames
